# The use and drawbacks of risk-grouping in prediction models

**DOI:** 10.1186/s12916-019-1485-4

**Published:** 2020-02-03

**Authors:** Kay See Tan, Melissa Assel

**Affiliations:** Department of Epidemiology and Biostatistics, Memorial Sloan Kettering Cancer Center, 485 Lexington Avenue, 2nd Floor, New York, NY USA

**Keywords:** Prognostic model, Validation, Clinical trials, Competing risks, Risk categorization, Decision curve analysis, Nomogram

## Background

The goal to provide patients with accurate prognosis has motivated the development of prediction models across different diseases. In renal cell carcinoma (RCC), various prognostic models have been published to guide patient care and facilitate selection into clinical trials, such as the University of California Los Angeles Integrated Scoring System (UISS) [1] to predict overall survival, and the Leibovich model [2] to predict disease-free survival.

Recently, Klatte et al. [3] developed the VENUSS model to predict outcomes following curative surgery for non-metastatic RCC. Unlike the UISS and Leibovich models, the VENUSS model predicts recurrence among papillary RCC, and appropriately utilized a competing risk approach [4] to account for competing events (e.g., deaths without recurrence) in the analyses. The final multivariable model was converted into a simplified scoring algorithm, and then, based on cumulative incidence of recurrence, further categorized into low, intermediate and high-risk groups. The authors then evaluated the predictive performance of the risk groups: after estimating a new model based on dummy variables for each risk group, multiple predictive performance metrics (c-index, calibration plots, decision curve analysis) were assessed. The authors concluded that the VENUSS model may be superior to standard models.

In their study, Klatte et al. [3] demonstrated the clinical importance of VENUSS risk groups to define eligibility in clinical trials. However, when assessing individual patient risks, we argue that the perceived benefit of a user-friendly risk-grouping approach is outweighed by the loss of precision in risk estimation, particularly in the era of personalized medicine.

Risk-grouping provides a qualitative assessment of prognosis by identifying patients at different risk levels for an event of interest. Risk-grouping can also provide a crude estimate of risk using simple back-of-the-envelope calculations, and have thus gained popularity in clinical practice. In the VENUSS model [3], simplified risk scores (0–11 points) are first derived by summing integer points assigned to each level of five clinical characteristics found to be associated with recurrence. Based on cumulative incidence of recurrence curves, the authors then grouped the scores to define low (0–2 points), intermediate (3–5 points) and high (≥6 points) risk groups, corresponding to 5-year cumulative incidence of recurrence of 2.9, 15.4 and 54.5%, respectively. Physicians can thus utilize VENUSS risk groups for prognostic stratification in adjuvant trials.

### Risk-grouping leads to loss of information

Categorizing predictions into risk groups implies that the risks (or probabilities) are identical for all individuals within each group, resulting in the loss of granularity in risk estimates. For example, the 5-year cumulative incidence of recurrence in the ‘intermediate risk’ group may range between 10 and 25%, depending on VENUSS scores of 3, 4, or 5 [3]. This crude grouping results in a loss of information crucial for individualized disease management [4].

### Precise risk estimation can guide personalized treatment

A clear benefit to prediction models is that patient-specific risk predictions can be directly obtained to guide patient care. Informed treatment decision-making requires the understanding of a patient’s ‘threshold probability’ – the critical point at which the expected benefit of the treatment equals the expected benefit of avoiding the treatment – and above which would prompt a patient to opt for adjuvant treatments. A cancer-averse patient may opt for adjuvant treatment at a predicted 5-year recurrence rate of 5%, whereas a treatment-averse patient may only do so when the risk of recurrence is above 35%. Using a decision curve, physicians can demonstrate the net benefit of receiving adjuvant treatment at various threshold probabilities [5].

The VENUSS study [3] presented multiple decision curves, but their utility in providing patient-specific risks is limited because such risk-grouping de-emphasizes the variability in threshold probabilities. The three predefined risk groups produced exactly three discrete points instead of a continuous curve reflecting a range of potential threshold probabilities. Consider a scenario in which a patient contemplates whether to undergo adjuvant treatment, where an applicable threshold probability for that decision ranges between 10 and 20% for the outcome of recurrence at 5 years: all patients in the VENUSS intermediate risk group (group-based risk of 15.4% at 5 years) would have been recommended for adjuvant treatment. However, depending on where they fall within the risk group, patients may have made a different decision if they were provided with a specific recurrence probability at a landmark time instead of the VENUSS group. Thus, the precision of risk predictions enhances the shared decision-making process between patients and physicians to incorporate individual risk tolerance.

### Generating precise risk estimates in the modern era

Instead of risk groups, the predicted probability of recurrence at a clinically relevant timepoint should be utilized for individualized patient care. The latter is more accurate, and can be derived directly from the prediction models. Previously, simplified scoring algorithms were favored because it was tedious and complicated to estimate precise outcome probabilities for time-to-event outcomes. This challenge has now been overcome by technology: prediction models can be translated into nomograms for publication [6], or transformed into web-based calculators [7]. By inputting specific patient characteristics, these open-access prediction tools can provide patient-specific predictions of cancer outcomes across different diseases, such as the 5-year recurrence-free probability following surgery for RCC [7].

## Summary and recommendations

The current VENUSS risk grouping is valuable to define cohorts for clinical studies; however, to use VENUSS in the context of estimating patient-specific risk, the following recommendations must be considered. First, following the TRIPOD guidelines [8], the VENUSS study should provide adequate detail (cumulative baseline hazards, nomograms or web-based calculators) to allow calculations of patient-specific risks rather than only group-based risks. Second, any simplification of a developed prediction model is susceptible to some loss of predictive accuracy because of rounding [9]: we recommend formal validation of the VENUSS model using original model regression coefficients and thorough reporting of the predictive performance metrics before and after simplification of the scoring system [8, 10]. Third, comparisons with other RCC models must be conducted on the basis of validating the original model coefficients rather than risk groups. Addressing these recommendations would establish the validity of the VENUSS model for patient-specific risk estimation.

## Data Availability

Not applicable.
